# Validação do indicador do consumo do álcool *per capita* (APC) no Brasil

**DOI:** 10.1590/0102-311XPT207024

**Published:** 2025-08-25

**Authors:** Paula Carvalho de Freitas, Patricia Pereira Vasconcelos de Oliveira, Oscar Geovanny Enriquez Martinez, Ísis Eloah Machado, Inês Fronteira, Deborah Carvalho Malta, Paulo de Lyz Girou Martins Ferrinho

**Affiliations:** 1 Instituto de Higiene e Medicina Tropical, Universidade NOVA de Lisboa, Lisboa, Portugal.; 2 Ministério da Saúde, Brasília, Brasil.; 3 Universidade Federal do Espírito Santo, Vitória, Brasil.; 4 Escola de Medicina, Universidade Federal de Ouro Preto, Ouro Preto, Brasil.; 5 Escola Nacional de Saúde Pública, Universidade NOVA de Lisboa, Lisboa, Portugal.; 6 Escola de Enfermagem, Universidade Federal de Minas Gerais, Belo Horizonte, Brasil.

**Keywords:** Consumo de Bebidas Alcoólicas, Desenvolvimento Sustentável, Indicadores de Saúde, Estudos de Validação, Alcohol Drinking, Sustainable Development, Health Status, Validation Studies, Consumo de Bebidas Alcohólicas, Desarrollo Sostenible, Indicadores de Salud, Estudios de Validación

## Abstract

O consumo de álcool é um importante fator de risco para a saúde. A redução em 10% do consumo de álcool puro em litros *per capita* (APC) é uma das metas dos Objetivos do Desenvolvimento Sustentável (ODS). O indicador foi desenvolvido com dados nacionais e publicado em painel governamental (APC-Brasil). Este estudo tem como objetivo validar o método de cálculo do indicador APC, utilizando dados nacionais, para monitoramento dos ODS. Realizou-se validação de face do indicador por meio da pactuação da ficha de metadados por consenso de especialistas e validação externa por comparação com indicadores nacionais de consumo de álcool e o APC usado pela Organização Mundial da Saúde (OMS), utilizando análise da série temporal, correlação linear e diferença de médias. A ficha de metadados foi validada por especialistas. A análise dos indicadores para a validação externa demonstrou tendência estável do APC-Brasil, em contraposição à tendência de redução do APC-OMS; tendência crescente anual de 1,4% do consumo regular e de 1,2% para o beber episódico pesado e para a frequência de consumo entre 3 e 4 vezes na semana, redução de 4,7% na frequência de consumo diário; aumento de vendas de cerveja (2,3%) e vinho (3,5%) e correlação positiva do APC-Brasil com a taxa de mortalidade de pessoas com 15 anos de idade ou mais por causas plenamente atribuíveis ao consumo de álcool. O APC-Brasil foi validado. As comparações entre os indicadores demonstram a adequação do APC-Brasil para refletir a realidade nacional do nível de consumo de álcool da população, permitindo o acionamento de estratégias com vista à redução da carga desse consumo no Brasil.

## Abstract (en)

Alcohol consumption is an important health-related risk factor. A 10% reduction in pure alcohol consumption in liters per capita (APC) is one of the targets of the Sustainable Development Goals (SDGs). This indicator was developed using national data and published in a governmental panel (APC-Brazil). The aim of the present study was to validate the calculation method of the APC indicator using national data for monitoring SDGs. Face validation of the indicator was performed using a metadata form created through a consensus of experts. External validation was ensured by comparing the indicator to national alcohol consumption indicators and the APC used by the World Health Organization (WHO), employing time series analysis, linear correlation, and difference of means. The metadata form was validated by the experts. The analysis of indicators for external validation demonstrated a stable trend in the APC-Brazil in contrast to the downward trend in the APC-WHO; an annual upward trend of 1.4% in regular consumption and 1.2% in binge drinking and intake frequency between three to four times a week; a 4.7% reduction in the frequency of daily intake; an increase in beer (2.3%) and wine (3.5%) sales; and a positive correlation between the APC-Brazil and the mortality rate for individuals 15 years of age or older due to causes fully attributable to alcohol consumption. The APC-Brazil was validated. The comparisons of the indicators demonstrate the suitability of the APC-Brazil in reflecting national alcohol consumption patterns in the population, enabling the implementation of strategies aimed at reducing the burden of this consumption in Brazil.

## Abstract (es)

El consumo de alcohol es un importante factor de riesgo para la salud. La reducción del 10% en el consumo de alcohol puro en litros per cápita (APC) es una de las metas de los Objetivos de Desarrollo Sostenible (ODS). El indicador se elaboró con datos nacionales y se publicó en un panel gubernamental (APC-Brasil). El objetivo de este estudio es validar el método de cálculo del indicador APC, utilizando datos nacionales, para el seguimiento de los ODS. Se realizó una validación aparente del indicador mediante la aprobación de la ficha de metadatos por consenso de expertos y una validación externa por comparación con indicadores nacionales de consumo de alcohol y el APC utilizado por la Organización Mundial de la Salud (OMS), empleando análisis de serie temporal, correlación lineal y diferencia de medias. La ficha de metadatos fue validada por expertos. El análisis de los indicadores para la validación externa mostró una tendencia estable del APC-Brasil, en contraste con la tendencia a la baja del APC-OMS; un incremento anual del 1,4% en el consumo habitual y del 1,2% en el consumo episódico intensivo y en la frecuencia de consumo entre 3 y 4 veces por semana; una reducción del 4,7% en la frecuencia de consumo diario; un aumento de las ventas de cerveza (2,3%) y vino (3,5%); y una correlación positiva entre el APC-Brasil y la tasa de mortalidad en personas de 15 años o más por causas totalmente atribuibles al consumo de alcohol. El APC-Brasil fue validado. Las comparaciones entre los indicadores demuestran la idoneidad del APC-Brasil para reflejar la realidad nacional del nivel de consumo de alcohol de la población, lo que permite poner en marcha estrategias destinadas a reducir este consumo en Brasil.

## Introdução

O consumo de álcool é um importante fator de risco para o desenvolvimento de doenças e para mortalidade ^1^ ^,^ ^2^. No Brasil, foi o sétimo fator de risco para mortalidade e o quinto para incapacidades, sendo responsável por 3% de todos os óbitos e 3,5% dos anos de vida ajustados por incapacidade (*disability-adjusted life year* - DALYs) em 2021 ^3^. Nesse mesmo ano foram registrados cerca de 20 mil óbitos, considerando as causas diretamente atribuíveis ao consumo ^4^ ^,^ ^5^ ^,^ ^6^.

Além das implicações individuais, familiares e/ou sociais para a saúde, o consumo de álcool também tem um impacto significativo na saúde pública devido aos custos, diretos e indiretos, associados aos sistemas de saúde e à sociedade em geral ^7^. Estes são associados às bebidas consumidas, tratamento médico, hospitalização, reabilitação, perda de produtividade no trabalho e à justiça criminal ^8^. No Brasil, em 2019, o consumo de bebidas alcoólicas gerou um custo de R$ 18,8 bilhões. Destes, R$ 1,1 bilhão foi devido às internações e aos procedimentos ambulatoriais no Sistema Único de Saúde (SUS) ^9^.

Para mitigar os danos causados pelo álcool, é necessário o desenvolvimento de políticas públicas e ações estratégicas, cujo progresso e efetividade são monitorados por meio de indicadores, comumente provenientes de pesquisas de base populacionais e de dados administrativos ^10^ ^,^ ^11^. Entretanto, há uma gama de indicadores disponíveis e que trazem diferentes informações, sendo necessário, para uma avaliação de cenário epidemiológico, levar em conta suas especificidades, inclusive para fins de comparabilidade e reprodutibilidade ^11^.

A Organização Mundial da Saúde (OMS) traz em seu painel web Sistema Global de Informação sobre Álcool e Saúde (*Global Information System on Alcohol and Health* - GISAH) uma série de indicadores para monitoramento do consumo de álcool ^12^. Nesse contexto, pesquisadores ressaltam que os melhores indicadores para avaliar o consumo de álcool de uma população são: a prevalência do beber episódico pesado, prevalência de abstêmios e o álcool consumido *per capita* (APC) ^13^ ^,^ ^14^ ^,^ ^15^, preferencialmente com dados nacionais, como já calculado pelo Brasil ^16^. Ainda, destacam que o melhor indicador para avaliar o nível de consumo de álcool populacional é o APC (em litros de álcool puro) ^13^ ^,^ ^14^.

O mesmo entendimento é encontrado nos mandatos internacionais como os Objetivos do Desenvolvimento Sustentável (ODS) e o Plano de Ação Global para o Álcool 2022-2030, que trazem como indicadores-chave o APC e a prevalência de consumo episódico pesado para o monitoramento do progresso das metas pactuadas ^17^ ^,^ ^18^. No Brasil, o *Plano de Ações Estratégicas para o Enfrentamento das Doenças Crônicas e Agravos Não Transmissíveis no Brasil 2021-2030* (Plano de Dant) traz como indicador o beber episódico pesado ^19^.

Para medir e avaliar de maneira precisa o impacto do álcool na saúde pública, é essencial ter indicadores confiáveis e validados ^20^. No contexto dos indicadores dos ODS, caso do indicador APC, a validação da ficha de metadados deve ser realizada em conjunto com o instituto de estatística nacional, no caso do Brasil, o Instituto Brasileiro de Geografia e Estatística (IBGE) ^16^.

A validação externa é igualmente importante na análise dos indicadores de saúde. Isso envolve a comparação dos resultados obtidos por meio desses indicadores com outros dados confiáveis, como prevalência de consumo, registros de mortalidade, internações hospitalares e estudos epidemiológicos ^21^. Esta permite verificar se os indicadores são consistentes com outros dados disponíveis, garantindo que sejam próximos da realidade para a formulação de políticas de saúde.

Estabelecer um indicador válido que seja denominado “padrão ouro” é importante para o desenvolvimento de estudos epidemiológicos que deem conta de retratar de forma fiel o cenário de saúde da população. Este estudo tem como objetivo validar o método de cálculo do indicador APC, utilizando dados nacionais, para monitoramento dos ODS.

## Material e métodos

Trata-se de um estudo de validação de face e validação externa do indicador do álcool *per capita* nacional - APC-Brasil ^22^ ^,^ ^23^. Para o estudo, considerou-se o período de 2005 a 2020.

### Validação de face

A validação de face foi por meio de oficina de consenso, que se refere à apreciação e a pactuação entre os peritos que, de fato, o indicador mede aquilo que se pretende medir ^23^ ^,^ ^24^. A planilha com informações, fontes de dados e método de cálculo foi enviada previamente ao IBGE para verificar a conformidade, especialmente em relação à Pesquisa Industrial Anual - Produto (PIA-Produto) e às projeções populacionais ^25^ ^,^ ^26^.

A reunião contou com especialistas das seguintes instituições e quantitativos: IBGE (n = 6), Ministério da Saúde (n = 10), Organização Panamericana da Saúde (OPAS; n = 2), Universidade Federal de Minas Gerais (UFMG; n = 1) e Universidade Federal de Ouro Preto (UFOP; n = 1), selecionados com base em sua experiência, conhecimento técnico e capacidade de contribuir para o processo. Os convites foram enviados pela pesquisadora.

Para o IBGE, foram convidados os participantes dos grupos de trabalho da PIA-Produto e do grupo de trabalho dos ODS do IBGE; já no âmbito do Ministério da Saúde, foi convidado o grupo de trabalho dos ODS. O IBGE é o órgão responsável por calcular as estatísticas oficiais para o país, incluindo a validação dos indicadores dos ODS nacionais.

A OPAS foi convidada por ser responsável pela governança e gestão compartilhada do sistema nacional de saúde, de onde foram selecionados especialistas em políticas e estratégias de monitoramento e redução do consumo do álcool.

Da academia foram selecionadas professoras, doutoras e pessoas de referência em doenças crônicas não transmissíveis (DCNT) e seus fatores de risco, carga global de doenças e inquéritos em saúde pública, além de especialista em indicadores de consumo de álcool e consequências à saúde, inquéritos de saúde pública e carga global de doenças.

A oficina de consenso foi realizada em novembro de 2022. Foi utilizada a técnica do comitê tradicional ^27^, que consiste na discussão aberta do método de cálculo (e ficha de metadados) com especialistas, para se chegar a um consenso quanto a sua validade aparente. Nela, foi apresentado o método de cálculo do APC-Brasil e seus resultados, as fontes oficiais, o processo de extração dos dados, a série histórica disponível e a periodicidade de atualização do indicador. Foi também evidenciada a diferença dos dados obtidos pelo método de cálculo da OMS e do Brasil.

Procedeu-se à para apreciação e discussão em grupo com o objetivo de compartilhar as avaliações, identificar possíveis problemas, sugerir melhorias e validar o instrumento por consenso. Com o consentimento de todos os participantes, a reunião foi gravada para fins de registro e relatoria ^27^.

### Validação externa

Averiguou-se em que medida os resultados do indicador podem ser generalizados ou aplicados na população de estudo ^22^. Foi realizada análise mediante a comparação do APC-Brasil com indicadores nacionais sobre bebidas alcoólicas como: (i) consumo proveniente de inquéritos populacionais; (ii) vendas de bebidas alcoólicas; (iii) taxa de mortalidade plenamente atribuível ao consumo de álcool; e (iv) resultados do indicador APC calculado pela OMS.

#### Validação externa mediante a comparação com inquéritos populacionais

Para a análise de correlação dos indicadores de consumo, foram utilizados dados da pesquisa *Vigilância de Fatores de Risco e Proteção para Doenças Crônicas não Transmissíveis por Inquérito Telefônico* (Vigitel) ^28^ e da *Pesquisa Nacional de Saúde* (PNS) ^29^. Do Vigitel, foram utilizados os indicadores de prevalência de consumo de bebidas alcoólicas (aqueles que responderam “sim” à pergunta “O Sr(a) costuma beber álcool?”); e da frequência de consumo. Dentre as pessoas que referiram ter o costume de consumir álcool, aquelas que indicaram a frequência de consumo na pergunta “Com que frequência o(a) Sr(a) costuma consumir alguma bebida alcoólica?” foram codificadas como: 1, se a frequência for de um a dois dias por semana; 2 se for de três a quatro dias por semana; 3 se for de cinco a seis dias; e 4 para todos os dias.

Também foi incluído o indicador da prevalência de consumo episódico pesado (considerando quem respondeu sim às perguntas “Nos últimos 30 dias, o(a) Sr(a) chegou a consumir quatro ou mais doses de bebida alcoólica em uma única ocasião?” ou “Nos últimos 30 dias, o(a) Sr(a) chegou a consumir cinco ou mais doses de bebida alcoólica em uma única ocasião?”).

Da PNS (2013 e 2019) ^29^ foram utilizadas as perguntas: “Com que frequência o(a) sr.(a) costuma consumir alguma bebida alcoólica?” com as opções de resposta de 1 para “não bebo nunca”, 2 para “menos de uma vez por mês” e 3 “para uma vez por mês ou mais”; “Quantos dias por semana o(a) Sr(a) costuma consumir alguma bebida alcoólica?”, com as opções de reposta do número de dias (variável contínua) ou nunca ou menos de uma vez por semana; e “Em geral, no dia que o(a) Sr(a) bebe, quantas doses de bebida alcoólica o(a) Sr.a) consome?”, com número de doses de 1 a 98. Dentre as pessoas que referiram ter o costume de consumir álcool, foram consideradas aquelas que indicaram a frequência de consumo nas respostas.

O detalhamento sobre o método das pesquisas e da formação dos indicadores do Vigitel e da PNS pode ser encontrado em publicações específicas ^28^ ^,^ ^30^.

#### Validação externa mediante comparação com dados de vendas de bebidas alcoólicas

Os dados de volume (em litros) de vendas de bebidas alcoólicas no período de 2005 a 2020 foram extraídos da PIA-Produto, realizada pelo IBGE, disponíveis no Sistema IBGE de Recuperação Automática (SIDRA), que traz produção e vendas dos produtos e/ou serviços industriais, segundo as classes de atividades e os produtos.

Foram utilizadas as tabelas 5806 para os dados de 2005 a 2013 e 7752 de 2014 a 2020 ^31^. Foi selecionada a quantidade vendida, sendo todos os volumes padronizados para litros. Em classes de atividades industriais e produtos, foram utilizadas as categorias de bebidas alcoólicas seguindo a Nomenclatura Comum do Mercosul (NCM) ^16^.

#### Validação externa mediante comparação com dados de mortalidade

A mortalidade por faixa etária foi extraída do Sistema de Informação sobre Mortalidade (SIM) do Ministério da Saúde ^4^. O SIM é um sistema de vigilância epidemiológica nacional que tem como finalidade reunir dados quantitativos e qualitativos sobre óbitos ocorridos no Brasil e fornecer informações sobre mortalidade para todas as instâncias do sistema de saúde. Os dados estão disponíveis online por meio do Departamento de Informática do SUS (DATASUS) ^4^.

Os dados de mortalidade atribuível ao consumo de álcool foram selecionados com base nos códigos da 10ª versão da Classificação Internacional de Doenças (CID-10) onde o álcool aparece como causa necessária ^32^, no período de 2005 a 2020, por faixa etária. A taxa de mortalidade padronizada foi calculada utilizando o número de óbitos extraídos do SIM e o dado de população das projeções de população do IBGE ^26^. Foi considerada a faixa etária de 15 anos de idade ou mais, em consonância com a faixa etária calculada pelo indicador.

#### Validação externa mediante a comparação com o APC calculado pela OMS (APC-OMS)

Os dados do APC, já calculados pela OMS, foram extraídos do painel GISAH, utilizando-se o indicador APC total - Brasil, no período de 2005 a 2020. A análise de tendência desses dados teve como objetivo verificar sua correspondência com os indicadores nacionais.

### Técnicas estatísticas utilizadas na validação externa

As técnicas estatísticas utilizadas incluem a aplicação de testes de correlação de Pearson e a análise de séries temporais.

#### Análise de séries temporais

A análise de séries temporais é uma ferramenta valiosa na avaliação do consumo de álcool como indicador de saúde pública. Ela permite monitorar as tendências ao longo do tempo, identificar flutuações e avaliar o impacto de intervenções ou políticas específicas na redução ou aumento do consumo de álcool ^33^.

Para a análise das séries temporais, os dados de consumo de álcool (APC-Brasil, APC-OMS, prevalência, frequências, vendas e mortalidade atribuível ao álcool) foram transformados em logaritmos (log10). Realizou-se a regressão de Prais-Winsten com o indicador como variável desfecho e o ano como variável explicativa. Modelos com valor de p < 0,05 foram considerados com tendência não estacionária. A tendência de aumento ou decréscimo foi avaliada pelo valor de beta (β) e foi calculado o percentual (%) de incremento anual ^33^ ^,^ ^34^. Essa técnica estima tendência e associação em séries temporais, considerando a dependência dos seus valores ^33^.

#### Correlações

O teste de correlação de Pearson foi usado para medir as correlações entre os indicadores nacionais com distribuição normal e o APC-Brasil, incluindo prevalência e consumo abusivo de álcool, frequências de consumo e volume de vendas. A técnica avalia a direção e força da associação entre variáveis, permitindo analisar sua dependência estatística ^35^.

Para a análise dos dados de taxa de mortalidade de pessoas com 15 ou mais anos de idade, foi utilizada a correlação de Spearman, pelos dados não apresentarem uma distribuição normal após teste de normalidade de Shapiro-Wilk ^34^ ^,^ ^35^. Foram considerados significativos valores de p < 0,05.

No caso da PNS (2013 e 2019), foi utilizado o teste t de Student para amostras independentes ^35^. Todos os dados foram analisados no software SPSS versão 29.0.1.1 (https://www.ibm.com/).

## Resultados

### Validação de face

Para a validação de face, a ficha de metadados foi preenchida com as informações do país (Quadro 1). Após análise e debate, os especialistas concluíram, por unanimidade, que os dados utilizados eram adequados para o cálculo do indicador, como preconizado pela Organização das Nações Unidas (ONU). Também relataram que a disponibilidade dos dados governamentais para o método de cálculo é adequada para que o indicador reflita a realidade do país de forma mais verossímil.

**Quadro 1:** Ficha de metadados validada.

OBJETIVO DO DESENVOLVIMENTO SUSTENTÁVEL (ODS 3)	Assegurar uma vida saudável e promover o bem-estar para todos, em todas as idades *		
Meta 3.5	Reforçar a prevenção e o tratamento do abuso de substâncias, incluindo o abuso de drogas entorpecentes e uso nocivo do álcool *.		
Nome do indicador 3.5.2	Consumo de álcool em litros de álcool puro per capita (com 15 anos ou mais) por ano *.		
Conceitos e definições	Quantidade de álcool ingerida *per capita*, em litros de álcool puro, considerando a população total com 15 anos ou mais de idade, por ano, baseado na produção nacional, importação (reimportação), exportação e volume de álcool (graduação) para cada categoria de bebida alcoólica (álcool registrado + não registrado), ajustado pelo consumo de turistas **. LIMITAÇÕES: (1) Os dados têm uma defasagem de dois anos. A série histórica deve ser corrigida anualmente, para o ano atual e os dois anos anteriores, conforme correção da PIA-Produto. (2) Apesar da possibilidade de a variação dos estoques ser um componente importante para bebidas alcoólicas, a equipe da PIA-Produto recomenda usar a quantidade produzida e não a quantidade vendida, que acaba sendo subestimada pela pesquisa. (3) A PIA só considera empresas produtores com 30 empregados ou mais ***.		
Fórmula de cálculo	{[(Produção de bebidas alcoólicas + importação - exportação)/população total de 15 anos ou mais] + consumo de álcool *per capita* não registrado}, ajustado pelo consumo de turistas **.		
Unidade de medida	Litros de álcool puro *per capita* *		
Variáveis que compõem o indicador, suas respectivas fontes e instituições produtoras	Variáveis *	Fontes *	Instituições *
	Produção de bebidas alcoólicas no país e categorias de produtos*	Pesquisa Industrial Anual - Produto (PIA-Produto) **	IBGE 2018 **
	Importação (re-importação) e exportação de bebidas alcoólicas, por quilograma líquido *	COMEX STAT **	Ministério da Indústria e Comércio Exterior **
	Consumo de álcool turistas *	Estatísticas de turistas da ONU *	ONU *
	Consumo de álcool não registrado *	Estatísticas da OMS *	OMS *
	População residente de 15 anos ou mais *	Projeção e Retroprojeção da População do Brasil por sexo e grupo de idade **	IBGE 2018 **
Abrangência geográfica	Nacional **		
Níveis de desagregação	Nenhum **		
População alvo	População de 15 anos ou mais **		
Periodicidade de atualização do indicador	Anual **		
Série histórica disponível	2007-2020 ***		
Instituição produtora	Ministério da Saúde **		
Contato	Departamento de Análise Epidemiológica e Vigilância de Doenças não Transmissíveis. E-mail: daent@saude.gov.br. Telefone: (61) 3315-7705 **		
Referências	Referência ao metadado internacional:	https://unstats.un.org/sdgs/metadata/files/Metadata-03-05-02.pdf **	

IBGE: Instituto Brasileiro de Geografia Estatística ; OMS: Organização Mundial da Saúde; ONU: Organização das Nações Unidas,

* Informações padronizadas provenientes da ficha de metadados dos Objetivos do Desenvolvimento Sustentável (ODS);

** Informações nacionais incluídas para a oficina de validação;

*** Informações alteradas/incluídas na oficina de validação.

Ressaltou-se a importância de evidenciar as limitações do indicador, como a defasagem de dois anos dos dados, prazo necessário para a disponibilização pública da PIA-Produto pelo IBGE. Além disso, o IBGE apontou que a série histórica deve ser corrigida anualmente para o ano atual e os dois anos anteriores, conforme a correção da PIA-Produto.

O IBGE recomendou usar a quantidade produzida, e não vendida, para bebidas alcoólicas, pois, apesar da possibilidade de a variação dos estoques ser um componente importante para bebidas alcoólicas, a quantidade vendida acaba sendo subestimada pela pesquisa. Além disso, a PIA-Produto é divulgada no meio do ano, período recomendado para atualizar o indicador.

### Validação externa

#### Inquéritos nacionais

**Figura 1:**
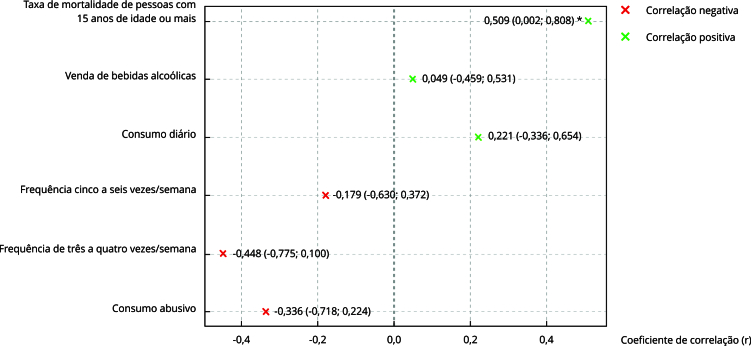
Correlações entre o APC-Brasil e os indicadores nacionais relacionados ao consumo de bebidas alcoólicas.

A Figura 1 apresenta as correlações entre o APC-Brasil e os indicadores nacionais de consumo referido de bebidas alcoólicas pela população. Observou-se correlação negativa entre o APC-Brasil e as prevalências de consumo: de três a quatro vezes/semana (r = -0,448); de cinco a seis vezes/semana (r = -0,179); e abusivo (r = -0,336); e correlação positiva entre o consumo diário (r = 0,221). Contudo, em nenhuma dessas correlações houve significância estatística (Figura 1).

Fonte: Sistema de Informação sobre Mortalidade ^4^, PIA-Produto ^31^ e Vigitel ^28^. * Correlação significativa (p < 0,05).

Ainda no que se refere aos indicadores nacionais do Vigitel, observou-se uma tendência anual crescente de 1,4% no consumo regular de bebidas alcóolicas (quem refere beber), e de 1,2% tanto no consumo abusivo (beber episódico pesado), quanto na frequência de consumo três a quatro vezes/semana. Em contrapartida, o consumo diário apresentou tendência de redução ao longo dos anos, com uma variação anual de -4,7%, conforme demonstrado na Tabela 1.

**Tabela 1:** Tendência indicadores de consumo de álcool: APC-Brasil ^22^ ^,^ ^23^, APC-OMS ^12^, Vigitel ^28^, taxa de mortalidade e vendas de bebidas alcoólicas, Brasil, 2005 a 2020.

Indicadores	Ano																Valor de p	Taxa (%) *
	2005	2006	2007	2008	2009	2010	2011	2012	2013	2014	2015	2016	2017	2018	2019	2020		
APC-Brasil **	8,4	8,4	8,4	8,4	8,4	8,5	8,6	8,4	8,3	8,1	7,9	7,6	7,4	7,3	7,3	7,5	0,051	-
APC-OMS **	9,0	12,0	9,7	10,2	10,4	10,2	11,3	10,3	9,4	9,2	9,2	9,7	9,2	9,4	9,8	9,8	0,019	-0,9
Beber episódico pesado ***	n/a	15,7	16,5	17,2	18,5	18,1	16,5	18,4	16,4	16,5	17,4	19,1	19,1	17,9	18,8	20,9	0,011	1,2
Refere beber ***	n/a	32,0	38,5	36,9	37,7	38,7	35,8	37,6	34,9	35,5	36,2	39,9	41,1	40,4	41,1	44,6	0,009	1,4
1 e 2 vezes *** ^#^	n/a	57,1	52,6	56,6	57,2	58,6	60,6	59,5	60,2	59,9	58,8	55,4	54,5	49,1	52,1	51,4	0,238	-
3 e 4 vezes *** ^#^	n/a	8,2	8,3	9,7	9,3	9,9	9,3	10,0	9,8	9,0	10,4	10,6	10,0	10,5	11,0	8,5	0,028	1,2
5 e 6 vezes *** ^#^	n/a	1,3	2,0	1,5	1,3	1,6	1,6	2,0	1,9	1,6	1,2	1,5	1,6	1,5	1,6	1,6	0,943	-
Diário*** ^#^	n/a	4,0	6,2	4,5	4,4	3,4	3,5	3,9	3,7	3,7	3,0	2,7	3,1	2,5	2,8	2,4	0,000	-4,7
Taxa de mortalidade ^## ###^	11,1	11,4	11,8	12,2	11,7	12,2	12.,6	12,2	12,2	11,6	11,5	11,3	11,1	11,0	10,6	12,2	0,82	-
Geral ^§^	10. 006	10.813	11.608	12. 209	13. 212	14. 297	14. 508	15.052	13.445	13.296	13. 504	13.287	12.375	13.160	14.552	13.817	0,06	-
Vinho ^§^	195	250	227	230	226	220	242	260	225	236	232	238	304	322	381	358	0,005	3,5
Cerveja ^§^	8.573	8.978	10.005	10.637	11.642	12.853	12.904	13.610	12.111	12.152	12.348	11.974	11.025	11.832	13.221	12.521	0,04	2,3
Cachaça ^§^	841	1.205	1.046	966	989	943	1.064	931	868	777	778	896	854	851	796	789	0,002	-1,8

APC: consumo de álcool *per capita*; OMS: Organização Mundial da Saúde; Vigitel: *Vigilância de Fatores de Risco e Proteção para Doenças Crônicas não Transmissíveis por Inquérito Telefônico.*

* Taxa de incremento médio anual, calculada pela fórmula [-1+10^β]×100, em que β é o coeficiente resultante da regressão de Prais-Winsten;

** Litros de álcool puro *per capita*;

*** Prevalência (%) segundo dados do Vigitel;

^#^ Frequência de consumo na semana;

^##^ Taxa por 100 mil habitantes, padronizada;

^###^ em pessoas com 15 anos de idade ou mais;

^§^ Volume de vendas em decâmetros (1.000L^3^).

Em relação aos dados da PNS, percebe-se que, apesar de o percentual de pessoas que referem beber uma vez ou mais por mês ser menor em 2013 (24%) que em 2019 (27%), as médias de consumo foram maiores na PNS de 2013 do que na de 2019. A frequência média de consumo de álcool por semana reduziu de 1,97 dias em 2013 para 1,86 dias em 2019 (t(38910) = 5.894; p < 0,01). O número médio de doses consumidas por ocasião também reduziu de 5,51 em 2013 para 4,4 em 2019 (t(23289) = 14.495; p < 0,01).

De maneira semelhante ao que foi evidenciado nos indicadores do Vigitel, os dados da PNS demonstram estabilidade na frequência de consumo de uma a duas vezes por semana. Também apontam para o aumento do consumo abusivo, sendo este em quatro ou mais doses para mulheres e cinco ou mais doses para homens numa mesma ocasião ^2^.

#### Vendas de bebidas alcoólicas

**Figura 2:**
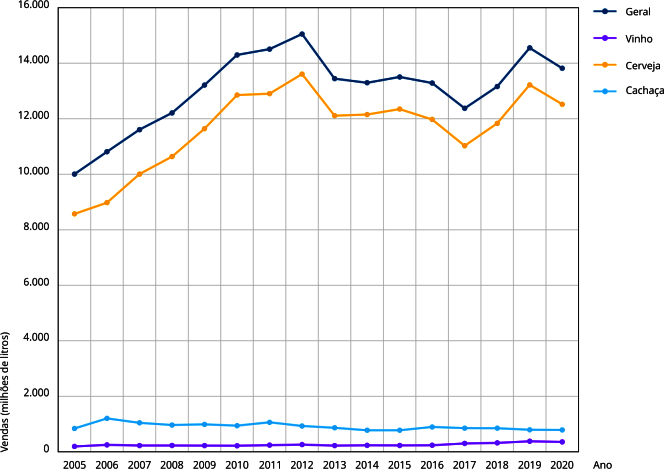
Vendas de bebidas alcoólicas no Brasil, 2005 a 2020.

Apesar da correlação positiva do APC-Brasil com as vendas de bebidas, esta não se mostrou estatisticamente significativa (Figura 1). O volume de vendas em quantidade de litros apresenta estabilidade, considerando todas as categorias de bebida (Tabela 1). Entretanto, dentre as bebidas alcoólicas mais consumidas no país ^36^, há aumento nas vendas de cerveja (2,3%) e vinho (3,5%) e um decréscimo nas vendas de cachaça (1,8%) (Figura 2).

Fonte: PIA-Produto ^31^.

#### Taxa de mortalidade

As taxas de mortalidade plenamente atribuível ao consumo de álcool na população de 15 ou mais anos de idade (Tabela 1) apresentaram correlação positiva moderada (p < 0,05 e r = 0,50) com o APC-Brasil (Figura 1). Porém, ao longo do período estudado, apresentou-se tendência de estabilidade.

#### Tendência do APC-OMS e do APC-Brasil

A análise de séries temporais revela uma taxa de redução anual de -0,9% no APC estimado pela OMS, contrastando com a tendência crescente observada na maioria dos indicadores nacionais, incluindo o APC-Brasil, que apresentou estabilidade ao longo do período analisado, com um brusco declínio de 2006 para 2007 (Tabela 1).

## Discussão

O indicador do APC-Brasil foi validado de forma aparente e por meio de validação externa com indicadores nacionais de monitoramento de consumo de álcool.

Para a validação de face, com o novo método de cálculo, procedeu-se ao preenchimento da ficha de metadados do indicador (Quadro 1). Na oficina realizada com especialistas, a ficha de metadados, o método de cálculo e as fontes de dados foram validados por consenso.

A equipe do IBGE avaliou os dados da PIA-Produto de 2005 a 2020 e identificou, em 2006, a entrada de grandes empresas na pesquisa, gerando um *outlier* na série. Por isso, o IBGE recomendou iniciar a série em 2007. Após essa revisão, os dados foram publicados oficialmente no painel online dos ODS e o indicador passou de Tier II (em produção) para Tier I (produzido).

As limitações incluem a defasagem de dois anos da PIA-Produto e a falta de dados sobre álcool não registrado no país. Esses dados poderiam ser obtidos futuramente com inquéritos nacionais que incluam uma pergunta sobre a origem da bebida. Contudo, o álcool falsificado, que representa cerca de 30% do mercado de álcool não registrado, permanece um desafio ^37^. Assim, recomenda-se a realização de inquéritos que captem o álcool não registrado para aprimorar o indicador do APC-Brasil.

No processo de validação externa, o resultado do APC-Brasil apresenta estabilidade para o período estudado, aproximando-se do que encontramos nas pesquisas nacionais de saúde, bem como na PIA-Produto ^25^ ^,^ ^29^.

A análise de tendência demonstrou-se uma etapa fundamental na validação externa dos indicadores, ao permitir uma compreensão mais acurada do panorama do consumo de álcool no contexto brasileiro com base em dados nacionais ^1^ ^,^ ^38^. A comparação entre os indicadores evidenciou limitações no APC-OMS, produzido com dados reportados pela indústria, que não captam adequadamente as especificidades locais, enquanto o novo indicador APC-Brasil apresentou maior aderência à realidade observada no país ^15^ ^,^ ^16^. De fato, a série temporal nacional revela uma tendência estável de consumo, em contraste com a tendência decrescente estimada pelos dados da OMS ^1^. Esse achado reforça a importância de utilizar indicadores construídos a partir de dados nacionais.

Adicionalmente, é importante a padronização das metodologias das pesquisas, de modo a garantir maior precisão na mensuração do consumo e na formulação e avaliação de políticas públicas eficazes. Ressalta-se que o estudo do consumo de álcool atualmente enfrenta desafios relevantes, como a heterogeneidade conceitual e metodológica entre os diferentes indicadores disponíveis, o que pode levar à subnotificação e ao desconhecimento da magnitude desse importante problema de saúde pública ^11^ ^,^ ^39^.

A série temporal do Vigitel demonstra o aumento das prevalências de referir beber álcool (32% em 2006 para 44,7% em 2020) e de consumo abusivo de álcool (15,7% em 2006 para 20,9% em 2020), além da frequência de consumo de três a quatro vezes por semana e entre as pessoas que tem o costume de beber. Destaca-se, ainda, que o aumento da tendência do consumo abusivo do álcool na população total teve forte influência do aumento desse padrão de consumo entre as mulheres (7,8% em 2006 para 16% em 2020), mantendo-se estável entre os homens de 2006 a 2020 ^28^.

A ausência de correlação entre os dados do Vigitel e o APC-Brasil pode ser devido a alguns fatores como a representatividade do inquérito, que traz dados das capitais e a subestimação de dados dos inquéritos referentes ao consumo de álcool ^11^ ^,^ ^38^.

A quantidade de litros vendida é coerente com o indicador APC-Brasil, mostrando estabilidade geral no período analisado. No entanto, houve aumento nas vendas de cerveja (2,3%) e vinho (3,5%) e uma queda nas vendas de cachaça (-1,8%). Isso sugere que o aumento nas vendas reflete maior consumo, como indicado nos inquéritos nacionais sobre consumo episódico pesado ^28^ ^,^ ^29^.

Apesar de apresentar correlação positiva entre venda e APC-Brasil, a ausência de significância estatística pode estar ligada ao fato dos dados da PIA-Produto estarem relacionados ao volume vendido pelo produtor aos revendedores e não ao consumidor final, contrariando o que se encontra na literatura internacional, em que apresentam forte correlação entre si. Além disso, outra hipótese é que o dado de venda pode subestimar o consumo total de álcool, uma vez que não contempla o álcool não registrado, que pode representar uma parcela substancial do consumo total de um país ^17^.

No Brasil, estudo realizado sobre o consumo abusivo de bebidas alcoólicas na população adulta brasileira entre 2013 e 2019 demonstrou que houve aumento da prevalência nesse período em todo o país ^2^. Os dados de prevalência de consumo de álcool demonstram o aumento deste durante o período estudado ^2^ ^,^ ^28^.

Entretanto, a estabilidade do APC-Brasil no período pode estar sendo impactada pela redução da frequência média de consumo de álcool por semana e do número médio de doses consumidas por ocasião de 2013 (1,97 dia e 5,51 doses) para 2019 (1,83 dias e 4,4 doses) ^29^ e também pela redução de 4,7% ao ano no consumo diário nas capitais brasileiras ^28^.

Outra hipótese pode estar relacionada à estabilidade do consumo abusivo na população masculina, à redução das frequências de consumo diário e à estabilidade na frequência de consumo cinco a seis dias na semana. Por outro lado, a tendência de redução apresentada pelo APC-OMS no período não condiz com a realidade do país ^16^, mostrando a importância de bases nacionais para captar o real consumo.

Inquéritos são essenciais para se monitorar a situação de saúde da população, entretanto, podem subestimar o consumo de álcool (método, alcance, periodicidade, viés de memória) ^33^ ^,^ ^40^. Essa subestimação foi verificada ao se comparar os resultados dos inquéritos aos dados de vendas e produção dos países. No caso das bebidas alcoólicas, destaca-se importância dos inquéritos e indicadores de consumo abusivo e de abstêmios como sendo componentes da análise do consumo no país ^15^.

Outro importante indicador para a análise de situação de saúde é a taxa de mortalidade ^40^. Ao verificar a taxa de mortalidade plenamente atribuível ao consumo de álcool, na população de 15 anos e mais, esta apresentou uma correlação positiva moderada com o APC-Brasil. Essa correlação também é reportada em estudos internacionais ^1^ ^,^ ^38^. A mortalidade atribuível está relacionada, especialmente, a condições crônicas ^32^. Assim, a taxa de mortalidade em pessoas a partir dos 15 anos de idade indica o consumo de álcool por jovens, tornando-se um importante problema de saúde no país. Isso é apresentado na *Pesquisa Nacional de Saúde do Escolar* (PeNSE 2019) ^41^, que demonstrou que 34,6% dos escolares de 13 a 17 anos tomaram a primeira dose de bebida alcoólica antes de completar 14 anos e 25,3% consumiram álcool nos últimos 30 dias anteriores à pesquisa.

Rehm et al. ^(^ ^13^ ^)^ estabelecem o APC como o indicador mais acurado e confiável para monitorar as tendências de consumo de álcool. Trata-se de um indicador-chave não só para entender a dinâmica do consumo de álcool, como para avaliar a efetividade de políticas públicas e seu alinhamento às metas dos ODS. O indicador tem a vantagem de ser econômico e de cálculo rápido, uma vez que não depende da realização de inquéritos. Além disso, é o indicador que melhor se correlaciona com a mortalidade e os danos à saúde ^15^.

Assim, o estudo buscou utilizar os melhores indicadores de consumo de álcool disponíveis, preconizados por pesquisadores e referenciados nos marcos pactuados internacionalmente, para a realização das correlações para a validação externa ^1^ ^,^ ^11^ ^,^ ^12^ ^,^ ^13^ ^,^ ^15^ ^,^ ^18^ ^,^ ^19^.

## Considerações finais

Entende-se que o APC-Brasil foi validado conforme proposto, com a validação da ficha de metadados e pelas correlações e análise das séries temporais. A correlação ser significativa estatisticamente apenas com a mortalidade reforça o achado em estudos internacionais e destaca o mencionado por pesquisadores sobre a necessidade de padronização de metodologias de indicadores de consumo, bem como de subestimação do consumo do álcool por meio de inquéritos populacionais.

Entretanto, ao analisar as tendências dos indicadores propostos, percebe-se que seu comportamento está alinhado ao do APC-Brasil e divergente do APC-OMS. Nesse sentido, pode-se inferir que o APC-Brasil informa de forma mais realista o cenário do consumo de álcool no país, devendo ser este o indicador utilizado, conforme disposto no painel governamental acima mencionado, uma vez que o uso de indicadores validados é importante para garantir a qualidade e a confiabilidade da informação.
